# Editorial Valedictory

**Published:** 1861-11

**Authors:** S. D. Gross


Editorial Valedictory.—With the present number closes the career of The North American Medico-Chirurgical Review; and, as its Senior, and, for some time past, its sole Editor, it becomes my duty to offer a few words in explanation of the circumstance.

The Review was commenced five years ago under auspices eminently favorable to success; the country was never in a more flourishing condition, and the only medical periodical then in existence in this city was the *American Journal of the Medical Sciences.* Under these circumstances, the Review, founded upon the *Philadelphia Medical Examiner* and the *Louisville Medical Review*, the latter commenced only a short time previously, made its appearance as a bi-monthly. Until the present national troubles arose, it enjoyed an uninterrupted prosperity, and I am quite safe in asserting that, but for these unhappy occurrences, which deeply affect all classes and ramifications of business, no one would have thought of suspending the publication of a work which has everywhere been received with favor.

It is a self-evident fact that no medical journal can long maintain itself without a large circulation, and hence, cut off as ours has been during the last six months from the Southern States, it would be unwise to attempt to prolong an existence which would only involve further expenditure. Whatever desire the Editors might have to continue the enterprise, they could not regard with indifference the interests of their publishers, who have, upon all occasions, responded, in the most prompt and generous manner, to every demand designed to promote the usefulness of the work. Our editorial relations, too, are materially changed. For the last three years, Dr. Richardson, one of the originators and planners of the journal, has been a resident in a distant city, with no leisure to afford me any substantial aid. In order to supply a loss which, to me, as well as to the readers of the journal, has been a serious one, my son, Dr. S. W. Gross, became associated with us in the editorial management of the work, and, although he kindly took upon himself all the drudgery which such an enterprise must always necessarily impose upon some one, yet the responsibility of the publication essentially devolved upon myself. He, too, has been absent for some time past; and I am, therefore, all things considered, rejoiced that the time has arrived when I shall be able to devote myself, untrammeled, to other and more congenial pursuits.

Five large volumes, comprising altogether nearly 6000 closely-printed pages, have been issued under our auspices, and it is confidently believed that there is no medical periodical in the English language which has furnished, within the same compass, and during the same period, a greater amount of valuable material. We are the more emboldened to say this because our own contributions have been comparatively few. Many of the reviews are characterized by marked ability and learning, while quite a number of the original articles would compare favorably with those of any medical journal in the world. The abstracts, generally prepared with much care, have been largely read and widely copied, both at home and abroad.

It would be invidious to single out from our list of contributors the names of particular writers, whose readiness to serve us has placed us under special obligations; but we should be unjust to our feelings if we omitted to refer, in this connection, to Dr. John Bell, whose writings are so well known to the profession, and whose prolific pen has so often enriched the pages of the Review. To Dr. Richard J. Dunglison my acknowledgments are due for important aid rendered me in bringing out the last three numbers.

In taking a retrospective glance at our labors, as Editors, we have the consolation to know that our pages have never, in a single instance, been sullied by the slightest personality, or willful injustice. While we have endeavored to discharge our multifarious and responsible duties with the strictest impartiality, we have never lost sight of the dignity of our office as public servants of the profession, or of the sacred trust confided to us by our subscribers. We lay aside the robes of office as white and as pure as they were when we entered upon our labors five years ago. If, in our dealings with authors, we have occasionally been severe, our verdict has always been tempered with mercy. Every just and laudable effort to improve our medical literature, and advance the interests of science, has invariably met with encouragement; while shallow forwardness and self-complacency have as invariably been rebuked.

The Review has, from its commencement to its close, paid liberally for every article, of the slightest import ance, that has appeared in its pages. Owing to this circumstance the work has not, commercially speaking, been remunerative, either to its conductors or its publishers, but they confidently believe that it has been productive of great good to others, and they, therefore, feel that their own labor has not been bestowed in vain.

The regularity with which the work has always appeared on the day of its promised publication must have struck all our readers. This has been due to the fact that we have never, at any time, been in want of material; on the contrary, we had, invariably, several communications lying over from one number to another, so that we were never obliged to hurry either our contributors, the printers, or ourselves. A journal conducted upon any other principle must be productive of immense annoyance, and often of great disappointment to all concerned.

To our contemporaries of the medical press we beg leave, in conclusion, to tender our most cordial acknowledgments for the courtesy and kindness which, with few exceptions, they have so uniformly extended to us. Our greatest regret in severing our editorial connection is, that we shall no longer have the benefit of their welcome exchanges.

S. D. GROSS.

